# The Use of *a priori* Information in ICA-Based Techniques for Real-Time fMRI: An Evaluation of Static/Dynamic and Spatial/Temporal Characteristics

**DOI:** 10.3389/fnhum.2013.00064

**Published:** 2013-03-11

**Authors:** Nicola Soldati, Vince D. Calhoun, Lorenzo Bruzzone, Jorge Jovicich

**Affiliations:** ^1^Center for Mind/Brain Sciences, University of TrentoTrento, Italy; ^2^Department of Electrical and Computer Engineering, University of New MexicoAlbuquerque, NM, USA; ^3^The Mind Research NetworkAlbuquerque, NM, USA; ^4^Department of Information Engineering and Computer Science, Faculty of Engineering, University of TrentoTrento, Italy; ^5^Department of Cognitive and Education Sciences, University of TrentoTrento, Italy

**Keywords:** real-time fMRI, ICA, *a priori* knowledge, dynamic monitoring, adaptive algorithms

## Abstract

Real-time brain functional MRI (rt-fMRI) allows *in vivo* non-invasive monitoring of neural networks. The use of multivariate data-driven analysis methods such as independent component analysis (ICA) offers an attractive trade-off between data interpretability and information extraction, and can be used during both task-based and rest experiments. The purpose of this study was to assess the effectiveness of different ICA-based procedures to monitor in real-time a target IC defined from a functional localizer which also used ICA. Four novel methods were implemented to monitor ongoing brain activity in a sliding window approach. The methods differed in the ways in which *a priori* information, derived from ICA algorithms, was used to monitor a target independent component (IC). We implemented four different algorithms, all based on ICA. One Back-projection method used ICA to derive static spatial information from the functional localizer, off-line, which was then back-projected dynamically during the real-time acquisition. The other three methods used real-time ICA algorithms that dynamically exploited temporal, spatial, or spatial-temporal priors during the real-time acquisition. The methods were evaluated by simulating a rt-fMRI experiment that used real fMRI data. The performance of each method was characterized by the spatial and/or temporal correlation with the target IC component monitored, computation time, and intrinsic stochastic variability of the algorithms. In this study the Back-projection method, which could monitor more than one IC of interest, outperformed the other methods. These results are consistent with a functional task that gives stable target ICs over time. The dynamic adaptation possibilities offered by the other ICA methods proposed may offer better performance than the Back-projection in conditions where the functional activation shows higher spatial and/or temporal variability.

## Introduction

Real-time fMRI (rt-fMRI) is an emerging neuroimaging tool based on the estimation of brain activity in real-time (typically around 1–2 s; Weiskopf et al., 2004, 2007; deCharms, 2008; LaConte, 2011). This tool can be used not only for overall monitoring of fMRI data quality (Weiskopf et al., 2007) but also for manipulating the cognitive state of the subject based on their own brain activity (Shibata et al., 2011). The neurofeedback approach has been used in various fields of cognitive neuroscience such as attention (Thompson et al., 2009) and emotion (Posse et al., 2003). Neurofeedback approaches have also been used with rt-fMRI in clinical research, such as the study of control of chronic pain (deCharms et al., 2005) and the control of craving (Chiu et al., 2010; Anderson et al., 2011).

Since its advent, rt-fMRI has had to face a number of technical challenges, mainly due to the computational load of the data analysis which directly competes against the goal of providing real-time feedback (i.e., <1 TR). However, recent technological advancements have provided a way to overcome this issue by making large scale computations possible even on standard platforms (Weiskopf et al., 2007; Weiskopf, 2012). These technical advances have enabled us to shift our focus of attention from technical issues to data analysis aspects.

The usual goal of a real-time system is to permit the identification and monitoring of an activity of interest during its ongoing development and actuation. The identification is defined as an initialization phase where the real-time analysis and derived spatial-temporal features to be monitored are defined, usually with a functional localizer (FL) or a classification training step (LaConte et al., 2007). The monitoring represents the execution of the on-line analysis of the event of interest and the real-time delivery of results that can eventually operate on the stimulation paradigm. From a conceptual point of view, it is thus possible to discriminate the identification and monitoring phases and to develop different algorithms and strategies to deal with them.

The initial and still most common analysis framework for rt-fMRI is based on univariate hypothesis-driven approaches, with adaptation of standard algorithms, such as the general linear model family (GLM), to the real-time domain (Cox et al., 1995; Gembris et al., 2000; Hinds et al., 2011). These methods are common mostly because they are associated with ease of interpretability and fast computation. In these approaches both the identification and monitoring phases are typically implemented using hemodynamic response-based models of the expected cognitive tasks and eventual nuisance variables taking place during the rt-fMRI experiment.

Another family of data analysis techniques is represented by the multivariate data-driven algorithms, which have shown a great capability of exploiting the full information content intrinsically present in the data to be analyzed without assuming the explicit shape or timing of the hemodynamic response to a stimulus (McKeown et al., 1998; Mouro-Miranda et al., 2005; Norman et al., 2006). The driving motivation behind these methods is that they allow characterizing functions that may not be detectable without exploiting both second order (variance) and higher-order statistics, thus relying on a greater amount of information. These properties make the multivariate data-driven techniques very appealing for use in the real-time domain. Within this concept several machine learning algorithms have been successfully adopted and exploited in the real-time data analysis framework. The most successful implementations are based on support vector machines (SVM; LaConte et al., 2007; Magland et al., 2011; Sitaram et al., 2011). SVM provides a powerful solution to a number of applications that are subject specific, at the cost of training the classifier and imposing some interpretability issues on the results. In this context, the two phases of the canonical rt-fMRI framework are represented by the two steps of a classifier, i.e., the first phase is the training of the classifier, and the second phase is the test or execution of the classifier (i.e., the classification itself).

In addition to SVM, independent component analysis (ICA), another multivariate data-driven technique, has proven to be very effective in fully exploiting the complete amount of information which is present in the data. ICA enables the extraction of knowledge other than that merely modeled in a classical univariate approach (Hyvrinen and Oja, 2000; Calhoun et al., 2001; Beckmann and Smith, 2004). Furthermore, ICA methods can also be applied in a series of problems for which univariate inference cannot offer a solution, i.e., in experiments that lack a regressor model to be adopted in the univariate analysis. This is the case for resting data analysis or also experiments with particular patient populations (Calhoun et al., 2009).

The idea of translating ICA properties to a real-time implementation was firstly proposed by Esposito et al. (2003) in a seminal paper and implemented as a plug-in in Turbo Brain Voyager software (Goebel, 2012). In this initial work the authors presented a FastICA based rt-fMRI analysis tool exploiting precise design choices and including an identification phase and a monitoring phase. The first identification phase solved the problem of ranking ICs of interest, i.e., a canonical univariate functional localizer step was implemented to define areas of interest. Other ways to solve the problem of ICs ranking could be represented by exploitation of expected characteristic features of the ICs of interest via a classifier (DeMartino et al., 2007). The second monitoring phase used on-line execution of FastICA (implemented in a sliding window fashion) for extracting different ICs. The ICs were ordered on the basis of their spatial overlap with the IC of interest, which in this case consisted of single-slice representation of motor activity derived from a finger tapping localizer.

The work presented by Esposito et al. (2003) was recently extended to evaluate the performance of 14 different ICA algorithms considering as additional variables the model order and different types of *a priori* knowledge (spatial/temporal; Soldati et al., 2013). This work showed that ICA algorithms such as EVD, amuse, jadeopac, and FastICA were suitable when implemented in the identification phase via a functional localizer since they performed well even without extensive use of *a priori* knowledge. It is interesting to note that FastICA algorithm represented a good trade-off and its performance was valid in both functional localizer and dynamic monitoring phases. Other algorithms like constrained ICA performed worse without *a priori* knowledge and may thus be more suited for the dynamic monitoring phase due to their ability to incorporate *a* *priori* knowledge. Such *a priori* knowledge may help guiding the algorithm to detect a specific target IC with higher priority over the other ICs present in the data. However, there are several types of prior information that are available including spatial domain, the temporal domain, or both, and any of these could be used in different ways (as constant references from a localizer or derived dynamically). It is however not clear how these various ways of using priors may affect the performance of the results both in terms of computation time and correlation to a reference optimal ICA. Moreover, the ICA algorithm (FastICA) is stochastic, which means that multiple repetitions of the analysis on the same dataset can give slightly different results, both in the spatial and temporal domains. The problem has been extensively discussed in the literature, with one of the main proposed solutions being based on multiple ICA runs and clustering of the obtained components, with the aim of reducing the issue of stochastic variability (Himberg et al., 2004). Such instabilities can be characterized by the standard deviation of the derived (STD) results (spatial and/or temporal) when the analysis is repeated multiple times on the same dataset. The STD can be considered as a stability performance parameter of the algorithm, lower STD algorithms corresponding to more stable ones. This parameter may be particularly relevant if different ICA-based algorithms are to be considered and compared for real-time fMRI, where the analysis is repeated dynamically during data acquisition.

This study extends previous work (Esposito et al., 2003) in two ways. Firstly, the target IC to be monitored dynamically is identified from a functional localizer using an ICA-based method instead of using a GLM of the hemodynamic response. This approach allows the full analysis pipeline to be multivariate and data-driven. Secondly, novel ICA-based algorithms are proposed that introduce different types of *a* *priori* knowledge for the dynamic monitoring of ongoing fMRI activity. The main goal of this study was to evaluate how these algorithms perform with respect to an off-line ICA analysis after the acquisition is complete. The *a priori* information considered was either temporal, spatial, or both spatial and temporal. In addition, the *a priori* information was considered both in its static version when derived from the functional localizer, as well as dynamic when estimated recursively as the sliding window progresses over the time course throughout the run. The different ICA-based analysis methods proposed here were tested by artificially simulating a real-time fMRI experiment using real fMRI data from a visual motor study (Calhoun et al., 2001). The original data used is unrelated to a real-time fMRI experiment, but was adopted because it is public and offers robust functional activation in well-known anatomical areas. The measures of performance to compare the various real-time methods were based on the following three metrics: (i) spatial and/or temporal correlation between the independent component (IC) estimated dynamically and the target IC derived from the localizer, (ii) computation time, and (iii) intrinsic stochastic variability of the algorithms as estimated from multiple analysis runs.

## Materials and Methods

### Dataset

One of the aims of this work was to test a variety of ICA implementations in a fashion which can be directly applied to real world conditions. For the simulation of the rt-fMRI experiment we used a dataset coming from a real publicly available fMRI experiment, with tasks that show robust activation in well-known brain networks. We chose to use the data that comes as part of the GIFT package (Calhoun and Adali, 2006) because the ICA characterization of the task-induced activation networks was extensively tested. The dataset is thus publicly available and in the release it is stated that The Johns Hopkins Institutional Review Board approved the protocol and all participants provided written informed consent.

#### Imaging parameters

Scans were acquired on a Philips NT 1.5-T scanner. A sagittal localizer scan was performed first, followed by a T1-weighted anatomic scan [repeat time (TR) = 500 ms, echo time (TE) = 30 ms, field of view = 24 cm, matrix = 256 × 256, slice thickness = 5 mm, gap = 0.5 mm] consisting of 18 slices through the entire brain including most of the cerebellum. Next, we acquired functional scans over the same 18 slices consisting of a single-shot, echoplanar scan (TR = 1 s, TE = 39 ms, field of view = 24 cm, matrix = 64 × 64, slice thickness = 5 mm, gap = 0.5 mm, flip angle = 90°) obtained consistently over a 3-min, 40-s period for a total of 220 scans. Ten “dummy” scans were performed at the beginning to allow for longitudinal equilibrium, after which the paradigm was automatically triggered to start by the scanner.

#### Experiment setup

The GIFT package contains three subjects example data-sets that employ a visuo-motor paradigm derived from other studies (Calhoun et al., 2001). The paradigm contains two identical but spatially offset, periodic, visual stimuli, shifted by 20 s from one another. The stimuli consisted of an 8 Hz reversing checker-board pattern presented for 15 s in the right visual hemi-field, followed by 5 s of a central asterisk fixation, followed by 15 s of checker-board presented to the left visual hemi-field, followed by 20 s of a central asterisk fixation. The 55 s set of events was repeated four times for a total of 220 s. The motor stimuli consisted of participants touching their thumb to each of their four fingers sequentially, back and forth, at a self-paced rate using the hand on the same side on which the visual stimulus is presented.

#### Pre-processing

The images were first corrected for timing differences between the slices using windowed Fourier interpolation to minimize the dependence upon the reference slice chosen. Next, the data were imported into the statistical parametric mapping software package, SPM99. Data were motion corrected, spatially smoothed with a 6 mm × 6 mm × 10 mm Gaussian kernel, and spatially normalized into the standard Montreal Neurologic Institute space. The data were slightly subsampled to 3 mm × 3 mm × 5 mm, resulting in 53 × 63 × 28 voxels.

In this study the pre-processing steps were not included as part of the real-time fMRI simulations for several reasons: (i) the pre-processed and not the raw data are publicly available as part of the GIFT package (Calhoun et al., 2001) thereby being a reference starting point for various analysis tools, (ii) these pre-processing steps can be performed in real-time as several review studies describe (LaConte, 2011; Caria et al., 2012; Maclaren et al., 2013), (iii) the focus of this simulation study was on the data-driven network characterization through various real-time algorithms. For these reasons, and to keep a manageable number of variables in this study we limit our simulations to the manipulation of real-time analyses that follow the standard pre-processing steps.

### Toolbox and PC

The entire simulation work was based on an in-house implementation with MATLAB (2010) of the tested algorithm based on the code of GIFT toolbox (Calhoun and Adali, 2006). Given the ICA algorithms code present in the toolbox, all the data analysis steps (presented in Materials and Methods section) were implemented in an automatic fashion to permit a testing routine to be run by varying parameters, techniques, *a* *priori* knowledge, and different subjects. The PC adopted to run the simulations was an Intel(R) Core(TM) i5 CPU M460 @ 2.53 GHz equipped with 6 GB of RAM and running a Windows 7 64-bit OS.

### ICA mathematical preliminaries

Since all the methods share a common core based on ICA principles, we briefly recall the main concept associated with the ICA. Let’s assume that we have a set of fMRI measurements *Y_i_* where *i* = 1, …, *v* is the index of voxels and each *Y_i_* is a vector of *y_ij_* elements, where *j* = 1, …, *t* is the index of time points. The entire dataset can thus be represented as a matrix *Y* of dimensions time points by voxels. Now, let’s assume that the signal measured in the dataset is generated by a subset of *n* underlying sources which are linearly mixed and summed up. This reflects in the following canonical formulation using the vector-matrix notation.

(1) Y=AX

where *Y* is the acquired data matrix of dimension equal to the number of time points by the number of voxels, *A* is the mixing matrix of dimension equal to the number of time points by the number of sources to be recovered and *X* is the matrix of the sources (i.e., ICs) of dimension the number of sources by the number of voxels. Each *jth* row of *Y* is a vector *y_ji=1:*v*_* representing an fMRI volume in a *jth* time point and is thus obtained by the linear weighted combination of hidden sources spatial maps *y_j_* = *a_j1_*x*_1_ +…+ *a_jn_x_n_*∀*_j_**. This means

(2) $$Y=\overset{n}{\underset{j=1}{\sum }}a_{j}x_{j}$$

Given this definition and assuming that the sources *x_n_* are mutually independent, it is possible to recover those hidden sources by computing an estimation of the unmixing matrix *W* = *A*^−1^ such that

(3) X=WY

is an estimate of the sources. The estimation of *W* can be obtained via different algorithms, leading to different ICA implementations with different properties and effectiveness (see Bell and Sejnowski, 1995 for details). In this paper the selection of FastICA (Hyvrinen and Oja, 2000) as the core ICA algorithm has been driven by a recent study that compared the performance of 14 different ICA algorithms, and found FastICA to be amongst the most stable ones (Soldati et al., 2013). The FastICA algorithm exploits the non-Gaussianity as a metric of independence of the sources. This means, in the simplified iterative algorithm for several units, that the estimation of *W* is obtained through the following steps

1. initialize randomly *W*
2. given $W=\frac{W}{\sqrt{∥WW^{T}∥}}$
3. repeat until convergence $W=\frac{2}{3}W-\frac{1}{2}WW^{T}W$ and step 1–3.

Other approximations of the solution can be obtained, but a detailed description of the methods to obtain FastICA decomposition is anyway beyond the scope of the present paper.

### Analysis framework

The purpose of our analysis was to perform an extensive comparison between the standard off-line ICA analysis and several novel on-line ICA methods. The main goals of this study were to evaluate the feasibility of on-line ICA and identify the best performing algorithm from those proposed. For the purpose of our rt-fMRI simulations we proceeded with three different stages: calculation of reference ICs for performance evaluation, calculation of the target ICs from a functional localizer, and estimation of a dynamic IC on real-time. The first stage used FastICA on the complete fMRI time series to identify spatial and temporal IC templates from the networks that were later monitored dynamically. These templates were derived from the full dataset so they were in this sense considered as gold standard references against which the dynamically extracted components were later compared for spatial-temporal accuracy evaluations. The second and third stages were more strictly related to the rt-fMRI simulations. The second stage simulated a functional localizer (FL) session by taking the first 60 TRs of the fMRI time series. FastICA was used on the simulated FL to extract target ICs that was later monitored dynamically. The third stage represented the real-time fMRI simulation, the on-line ICA decomposition that used the information coming from the simulated FL. This last stage of real-time ICA decomposition was performed using the different novel techniques proposed and described in the next subsections.

**Figure 1:**
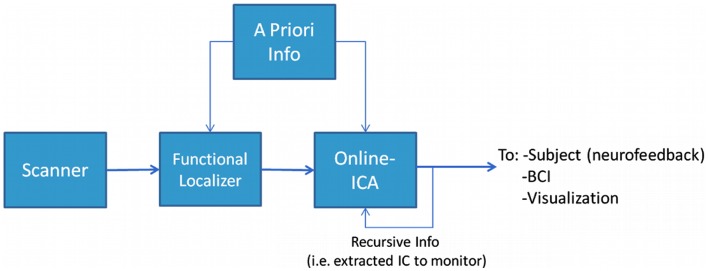
**The experimental design framework**. In this structure it is possible to identify two main phases (or steps). The first step is to identify what to monitor, i.e., performing a Functional Localization. This can be done with or without incorporating some kind of *a priori* knowledge. The second step is to monitor the phenomenon we identified in the previous step using a suitable on-line analysis method.

The proposed framework for performing rt-fMRI used a multivariate and data-driven approach schematically presented in Figure 1. The general structure and workflow can be outlined as follows: (1) The MR data acquired by the scanner was stored during acquisition and made available to the data analysis system as soon as the images were reconstructed. (2) At the beginning of the experiment a short period (typically about 5 min or less) was devoted to acquire data from a FL. In the proposed framework the FL data was analyzed using a blind (unconstrained) ICA algorithm to preserve the multivariate data-driven advantages. Others used univariate methods at this stage (Esposito et al., 2003). (3) An IC of interest was selected from the FL analysis, this IC became the data-informed multivariate ROI whose activity was meant to be monitored dynamically. (4) The IC of interest, along with possible *a priori* information, could be incorporated in the rt-ICA data analysis algorithm. The ICA algorithm used a sliding window approach and a blind source extraction (BSE) perspective to deliver results at each TR while updating the best match to the target component. This monitored component or other *a priori* knowledge was then provided recursively to the algorithm, which extracted the actual version of the monitored IC updated by the actual values of data. (5) The monitored IC could be used as in classical rt-fMRI paradigms for visualization, neurofeedback, or brain computer interfaces. The component selected in real-time was the one that has a spatial map which maximally correlates with the reference spatial map component identified during the Functional Localizer (FL) step. The spatial FL component corresponds in turn to the FL component whose temporal correlation with the timing of the paradigm was highest.

In this study two main different approaches were investigated to dynamically extract in real-time a target IC: static methods based on Back-projection and dynamic methods based on iteratively performed ICA. In particular, for the dynamic methods, FastICA, and constrained ICA were updated for a sliding window real-time fMRI implementation. The size of the sliding window was fixed and it was chosen based on previous work that systematically evaluated the performance of multiple ICA algorithms as function of window length amongst other variables (Soldati et al., 2013). This study showed that the sliding window length that gave the optimal trade-off between computational speed and spatial/temporal correlation with the results from the whole time course was approximately equal to the period of the behavioral task to be monitored. In our experiment this could be approximated to around 30 s, i.e., 15 TRs. It is worth noting that the size of this window, as pointed out in the discussion, was strongly related to the period of the behavior to be monitored.

#### Template creation and accuracy estimation

**Figure 2:**
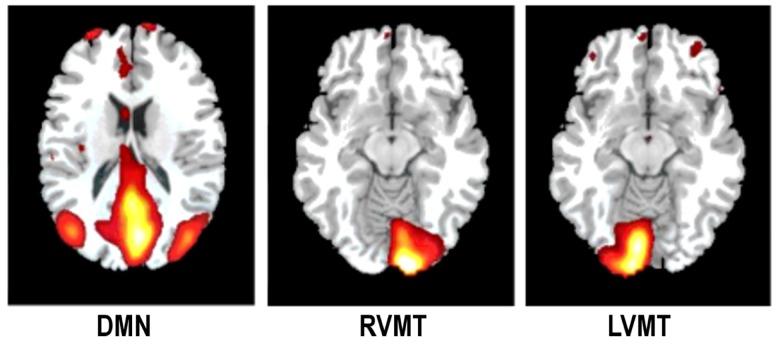
**The monitored ICs**. An illustrative example of the monitored ICs is reported. Spatial maps of ICs considered in the simulation are obtained from Group ICA 20 ICs. For ease of visualization only the relevant slices are reported here. First column depicts Default Mode Network (DMN). Second and third columns depict the two task related ICs, Right Visuo-Motor Task (RVMT) and Left Visuo-Motor Task (LVMT).

To estimate the accuracy of one technique in correctly describing a monitored IC at one arbitrary time point we generated task related network templates which represented the principal spatial and temporal characteristics of the ICs to be monitored during the simulation. These templates of task related ICs were thus taken into account as reference data to evaluate the quality of rt techniques. This evaluation was obtained via comparing the dynamically reconstructed ICs with these templates using temporal correlation and spatial overlap. To create the templates an ICA analysis was performed on the single subject level by considering all time points (i.e., 220 TR), but using FastICA with the same model order to be used in the on-line implementation (i.e., 5). Three different target ICs were manually selected to simulate their dynamic monitoring see Figure 2: two task related components (RVMT and LVMT) and the task-induced default mode network (DMN).

#### Functional localizer

In a rt-fMRI experiment the functional localizer could be used to identify the IC to be later dynamically monitored. For the analysis of the FL we considered the use of whole brain ICA to maintain a multivariate data-driven method. Previous simulations suggested that the ICA analysis of the FL data is most accurate when using algorithms such as evd, jadeopac, or FastICA (Soldati et al., 2013). We thus performed the FL applying the FastICA algorithm with a model order of 5, the same algorithm used in the template creation, to the first 60 TRs of the time series. With the application of FastICA as FL an unmixing matrix *W* was estimated of dimension 5 by number of time points. Each row of the matrix represented a time course of a hidden source, and the associated row of the *X* derived matrix represented the corresponding spatial map. The target IC to be monitored was then automatically selected as the one whose spatial map maximally correlated with the reference template and was monitored later dynamically with the on-line techniques. In this study three components were extracted from the FL for separate evaluations in the dynamic monitoring: the default mode network, the right, and left hemisphere visual motor networks activated by the cognitive task.

#### On-line techniques

In this section we present the main developed work, that is the methods implemented to perform the on-line monitoring of the sources. To simulate the on-line ICA analysis, coming after the FL, the rest of the time course (i.e., 220–60 TRs) was used to dynamically monitor the FL-derived target ICs using a sliding window approach. Given that the target was to properly exploit the *a priori* knowledge, different approaches to combine this knowledge and the ICA algorithms were developed. In a comprehensive perspective all the possible combinations were explored. Starting from the concept of sliding window ICA as it was presented by Esposito et al. (2003), more sophisticated and different methods were implemented. The target was to obtain an actual temporal value of the activation of interest and/or an actual spatial map of this component. Two main criteria were the guidelines in these implementations, that is the dynamic of the data and the type of *a priori* knowledge. The dynamic criterion means how much novel information is exploited and weighted into the on-line method, while the type of information exploited denote the nature of the *a* *priori* knowledge, i.e., temporal, spatial, or both. The implementation exploited state of the art ICA algorithms (FastICA, Constrained ICA) with the target of making the implementation easy to reply and distribute.

The following subsections present the details of the different on-line monitoring techniques proposed.

##### Static method: back-projection

The basic assumption behind this static method was that the brain activation of interest maintains its basic characteristics, in particular its spatial map (SM), relatively stable during the fMRI experiment. If this assumption holds, the spatial ICA performed in the FL step is enough to extract a precise representation of that spatial map that will be later tracked dynamically during the real-time experiment. ICA would be in fact able to create a space described by the directions of the extracted ICs that is fairly representative of the brain state during the performance of the task of interest. Given this assumption, the SM of an IC of interest obtained from the FL can be kept fixed and it should then be possible to simply back project each newly acquired volume of data into this space (i.e., onto the SM of the IC of interest) to be able to quantify the contribution of the new data to the brain activity of interest. This means that no ICA analysis must be performed in real-time, and the results will only depend on the ICA performed in the Functional Localizer session. This contribution will thus represent the time course of the IC. In more detail, the processing steps can be outlined as follows:

1. FastICA was used on the FL to estimate an unmixing matrix *W* = *A*^−1^ and thus the associated SM of the sources *X_nIC,v_* with nIC equal to the number of extracted components (5 in this case) and v equal to the number of voxels.
2. The SM of the desired component was then chosen as the source whose associated time course was the most correlated to the task of the FL, that is we have *X_nIC_=*sel,v**. The chosen spatial map was therefore an independent component computed by the FastICA algorithm, which gives it unitary variance and null mean value characteristics.
3. At this point, for each newly acquired volume *Y_ith_* of dimension one by number of voxels we could compute:
  (4) $$a_{ith}=Y_{i^{th}}X_{nIC=sel,v}^{†}$$
  where *a_ith_* is the actual single time value of the IC of interest and † denotes the pseudo-inverse. It is worth noting that this can be straightforwardly extended to cases in which multiple components are monitored simultaneously via parallelizing equation (4) for different SM or obtaining a meta-SM via combining different SM, i.e., *X* matrices.

##### Dynamic method: recursive temporally constrained

This algorithm is a direct extension of that used by Esposito et al. (2003). The main differences are that here it was applied to the whole brain and that the computation of *a priori* temporal knowledge was not model-driven, but it was rather data-driven and obtained with an approach based on the previously presented Back-projection method. The actual difference with the previously presented Back-projection method, in which the SM was static, was that we obtained an actual updated dynamic SM via iterative ICA computation. The details of the method are as follows:

1. From the FL a SM was obtained, which was used as in Back-projection method to obtain temporal *a priori* information in subsequent steps
2. During the experiment
  (a) using the Back-projection the time course of the brain activation was extracted
  (b) a FastICA algorithm with model order 5 and time window length 15 TR was applied to the data with a sliding window approach. The FastICA was temporally constrained using the *a* *priori* temporal constraint (obtained using the Back-projection) to initialize the mixing matrix *A*.

In practice a sliding window of dimension Δ was updated for each newly acquired volume *n* leading to a matrix *Y*_[*n*-Δ*,n*]*,v*_ of dimension Δ by number of voxels. This matrix and the SM obtained in the FL step (i.e., *X_nIC=sel,v_*) were used to extract a time course in a data-driven way in the same fashion as for the Back-projection algorithm, resulting in a time course *a_n_* of dimension *nTP* by one. With the actual data matrix *Y*_[*n*-*nTP,n*+*nTP*]*,v*_ and the time course *a_n_* it was then possible to apply FastICA to extract the actual SM of the component of interest. This was done by initializing the first entry of the *W* matrix with the inverse of *a_n_*, given that *W* = *A*^−1^, in the routine presented in the ICA mathematical preliminaries section. The result was the actual SM IC (i.e., *Xnew*) present in the data whose behavior was closest to the reference time course. In other words the extracted IC was constrained to be as close as possible to the reference one at the initial step, permitting a much more dynamic computation of the IC and thus update of the monitoring. In this approach the SM was dynamically updated each time a new volume was acquired.

##### Dynamic method: recursive spatially constrained

As in the previous method, also in this dynamic method, the on-line monitoring required a continuous update of the ICA decomposition matrix. There were two main differences with respect to the RTC method: (i) the ICA algorithm was a spatially constrained ICA (Lin et al., 2010), and (ii) the *a priori* knowledge was spatial instead of temporal. In this approach, the knowledge of an *a priori* SM of the IC of interest (obtained by the FL) permitted constraining the computation of the ICA algorithm. The constrained ICA algorithm was applied on time windows of data still of length 15 TR with a sliding window approach. The extracted IC, although based on newly acquired data, was forced to be spatially as close as possible to the spatial *a priori* given map (i.e., to the SM obtained during FL). This means that the dynamically extracted IC represented the SM of the brain activity of interest in the shape that was actually present in the novel data, thus dynamically updated. The associated time value was given by an approach similar to the Back-projection method but depending on the dynamically updated SM, i.e., given the new SM (i.e., *Xnew*), by computing $a_{ith}=Y_{ith}X_{new}^{†}$.

##### Dynamic method: recursive spatio-temporal method

This algorithm implemented the possibility of obtaining actual dynamic values from both the time course and the spatial map with two concatenated steps. This was obtained by combining the previous methods to obtain a fully updated on-line method based on the following steps:

1. Back project the actual data on the SM of FL, obtaining the actual value of time course in the FL space $a_{FL}=Y_{ith}X_{nIC=sel,v}^{†}$ (note that in the BP algorithm we assumed little or no difference between the template space and the actual subject space)
2. Apply the temporally constrained algorithm (i.e., initialize the *W* matrix exploiting the *a_FL_*) to obtain the SM (i.e., *X_sub_*) in the actual subject space (note that this is different from the FL space)
3. Apply the spatially constrained ICA with the SM in the actual subject space to obtain the actual time course value in the subject space, that is $a_{sub}=Y_{ith}X_{sub}^{†}$.

Adopting the described steps it was thus possible to obtain temporal and spatial values of the brain activation of interest fully exploiting the actual data, thus adapting to the dynamic changes which could occur, but keeping as a target the characteristics defined in the FL session.

### Variability effects from the stochastic nature of ICA

ICA methods (with some exceptions like the jade algorithm) are typically non-deterministic since there is a stochastic component in the analysis. This introduces variability each time the algorithm is run, which in turn can affect the computation time and the performance of the dynamic monitoring of a target IC. Such variability effects were investigated by repeating the analysis 10 times for each subject on the same data, and then computing the standard deviation across repeated trials for the mean correlation between dynamic and template spatial maps and temporal time course.

## Results

Using publicly available fMRI data from a previous experiment (Calhoun et al., 2001) we simulated a real-time acquisition in a sliding window approach to evaluate the performance of four implementations of ICA with different uses of *a* *priori* information: (i) Back-projection of constant spatial information derived from a functional localizer (BP), (ii) dynamic use of temporal (RTC), (iii) spatial (RSC), or (iv) spatio-temporal ICA constrained data (RSTC).

**Figure 3:**
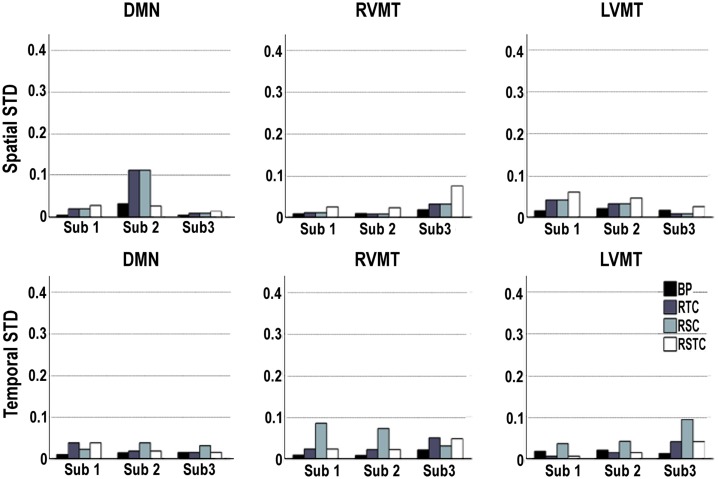
**Variability of dynamic tracking performance results due to the stochastic nature of ICA**. Performance is here represented by two metrics: spatial or temporal correlation between the template and the dynamically tracked IC, averaged along the time course. The calculations were repeated 10 times for each subject, for each of the three networks evaluated (default mode or DMN, right visual motor or RVMT, and left visual motor or LVMT), and for each ICA implementation (Back-projection or BP, temporally constrained or TC, spatially constrained or SC, spatio-temporal constrained or STC). The variability of the dynamic tracking performance results is expressed as the standard deviation across trials, per subject, brain network, and ICA implementation.

Given the stochastic nature of the ICA algorithms used, the variability of the spatial and temporal results was evaluated for each subject on each of the target networks (Default Mode Network (DMN), Right Visual Motor and Left Visual Motor Task related components (RVMT and LVMT respectively)) and for each of the four ICA implementations. The results showed in Figure 3 point out that stochastic effects can introduce variability in the performance of the IC order ranking accuracy up to 10%, sometimes producing large fluctuations. This behavior suggested that none of the four ICA implementations gave consistently the lowest sensitivity to fluctuations due to stochastic effects, although the BP method tended to be the lowest in 15 out of 18 cases.

**Figure 4:**
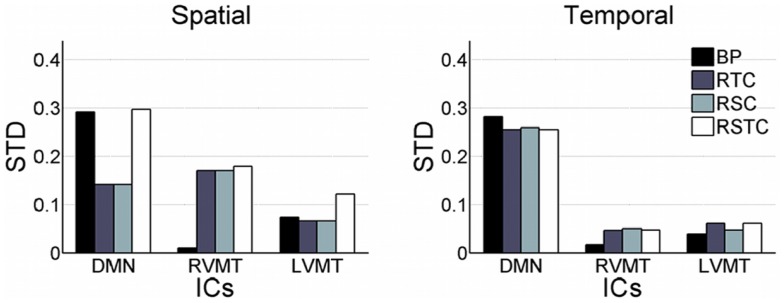
**Variability of dynamic tracking performance results due to subjects**. Similar to Figure 2, but here mean results across trials are used to compute variability across subjects, expressed as standard deviation. The subject variability is shown for each of the three networks evaluated (default mode or DMN, right visual motor or RVMT, and left visual motor or LVMT) and for each of the four ICA implementations (Back-projection or BP, temporally constrained or TC, spatially constrained or SC, spatio-temporal constrained or STC).

Proceeding with further analysis it was possible to focus on the evaluation of stability of results across subjects and across different monitored ICs, as presented in Figure 4. This figure reports the standard deviation across subjects of the mean (across trials, for each subject) spatial and temporal correlation for the monitored ICs.

**Figure 5:**
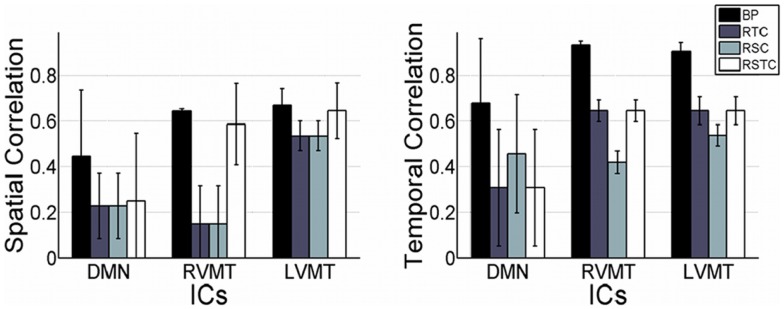
**Overall dynamic tracking performance in reconstructing ICs**. The mean and standard deviation performance results are shown across subjects and trials in terms of both spatial and temporal correlation with the template ICs. The results are shown for each of the three networks evaluated (default mode or DMN, right visual motor or RVMT, and left visual motor or LVMT) and for each of the four ICA implementations (Back-projection or BP, temporally constrained or RTC, spatially constrained or RSC, and spatio-temporal constrained or RSTC).

Finally the performance evaluation numbers were reported in Figure 5 and Table 1, in which one can see the spatial and temporal correlation between the reconstructed time courses and spatial maps of the monitored ICs and the reference templates of those ICs.

**Table 1:** **Performance**.

On-line method	CT[s]		
	DMN	RVMT	LVMT
Back-projection	0.0056 (0.0002)	0.0054 (0.0004)	0.0054 (0.0007)
Temporally constrained	2.3 (0.4)	2.1 (0.1)	2.0 (0.3)
Spatially constrained	0.119 (0.003)	0.1167 (0.0002)	0.1169 (0.0003)
Spatio-temporally constrained	0.123 (0.003)	0.1207 (0.0002)	0.1208 (0.0002)

*This table summarizes the performance results in terms of computational time (CT) from all the investigated rt-ICA techniques relative to the different monitored ICs. Mean values of updating CT are reported for simulations on three subjects and ten trials per subject. Standard deviations associated to mean values across subjects and trials are shown in parenthesis. The selected ICs to monitor were default mode network (DMN), right visuo-motor task (RVMT), and left visuo-motor task (LVMT)*.

The table reports the performances in terms of computational time necessary to update the actual value of time course or spatial maps for each new available data volume. This represents another critical issue of a real-time analysis.

The results show that the Back-projection method offered the highest performance both in terms of time course reconstruction (correlation value to the template time course was significant and quite high, around 0.9), and speed (computation of update value was far below the TR). This method was very fast and effective as long as the monitored IC had a strong and well defined behavior and/or it was well extracted in the FL, since it relied on an accurate description of the spatial behavior. The fluctuation reported in the figures represents error fluctuations in the FL phase which directly reflect in the Back-projection method. The dynamic methods offered comparable performances at cost of higher computational time (CT) (around 2 s for RTC). In particular the spatio-temporal method performed comparably in terms of CT to Back-projection, offering more variable performances in terms of reconstruction of spatial maps and time courses.

## Discussion

In the present work we presented and evaluated different methods to combine ICA-based algorithms for real-time fMRI. The motivation for this work was to investigate how the advantages of such multivariate data-driven based methods can be adapted to real-time fMRI applications, extending previous work (Esposito et al., 2003). One goal of this work was to simulate a realistic scenario fully based on ICA consisting of two essential steps. The first step was dedicated to identifying brain networks of interest from the ICA of a functional localizer. The second step consisted in dynamically monitoring a target IC (derived from the first step) with the use of different types of *a priori* knowledge in the computations. The *a priori* information considered ranged from static to dynamic, where spatial maps and time courses can be updated separately or together to give more weight to the dynamic monitoring of data within a pre-established time window in the fMRI time course. The incorporation of *a priori* information was motivated to address the challenge of identifying and keeping track of a specific IC of interest, despite all the other ICs that might be present in the data. This work therefore focused on evaluating different ways of using prior information about the target IC to monitor such that during the dynamic monitoring phase the target IC could be effectively detected with higher priority relatively to other possible ICs.

The ICA-based techniques presented for the on-line monitoring were characterized by different advantages and disadvantages. Overall findings confirmed two general expected features: (i) the dynamic monitoring performance was directly related to the strength of activation of the target IC identified in the functional localizer, stressing the importance of this first step, and (ii) as algorithms became more adaptive in the use of spatial and/or temporal priors in the dynamic monitoring, they introduced less stability in the performance results compared to off-line results. This reflected the intrinsic differences between static off-line analysis and dynamic one.

Back-projection is the only method presented for which the ICA is computed only once in the FL session, and not updated later during the on-line monitoring. This means that this method as implemented here is based on ICA since it depends on the quality of ICA performed in the FL session, but it is not a fundamentally ICA method. Back-projection could in principle also be used by defining a target brain network from the functional localizer with a standard general linear model that makes assumptions on the hemodynamic responses. The use of ICA, however, allowed the analysis to be fully data-driven (Esposito et al., 2003; Beckmann and Smith, 2004; Norman et al., 2006; Calhoun et al., 2009; Magland et al., 2011; Sitaram et al., 2011) and this represents an advantage in all those experimental designs where the classical ICA showed to be robust, as in all cases lacking a defined regressor. The Back-projection technique had the positive features of being stable in terms of lowest fluctuation across trial and subjects, very fast relative to the TR of fMRI data acquisition (since it just involves a matrix multiplication), conceptually simple, and being able to monitor more than one IC of interest. The main potential disadvantage of the Back-projection method was related to its non-adaptivity, since it assumed that the target IC of interest was always present with the same properties, i.e., a fixed spatial map was considered.

The temporally constrained ICA was more adaptive to data with respect to Back-projection. Even if similar to what was presented by Esposito et al. (2003) this method offered different characteristics. The main one was that the reference time course used as constraint was not obtained using a hemodynamic model, but it was extracted from the data in a multivariate data-driven way by the FL. Moreover in this method the reference time course was updated in a similar way to Back-projection, while the crucial difference was that the spatial map updates iteratively each time new data become available. A characteristic of the temporally constrained ICA was that the dynamic spatial map generated was derived from the time course used to initialize the ICA algorithm. This time course, being derived from the Back-projection of actual data on a static space (i.e., keeping the spatial map of the IC of interest fixed), was strictly related to the quality of the FL. For this reason the time course reconstructed was in the template space (i.e., FL space), while the spatial map was in the subject space, being obtained by exploiting the reconstructed time course as *a priori* knowledge during the application of the ICA algorithm. A limitation of the temporally constrained algorithm was that its mean computation time was more than one order of magnitude higher relative to all the other methods tested. This is due to the fact that the FastICA algorithm adopted in it, while performing generally lower than a TR, sometimes (around 2–3% of the times) got stuck in a local minimum thus increasing the time to perform the decomposition (in some cases from 1.5 upto 8 s). A possible solution to this would be to skip the updating of the information for those volumes which exceed a pre-determined temporal limit to update.

The spatially constrained ICA assumed a fixed spatial map of the IC of interest. This approach suffered from the small amount of data available for the decomposition. The main advantages included low computational time and low variability of the results, qualities that make it a good candidate for use in real-time experiments.

The combined implementation of spatial and temporal constrained ICA permitted a better description of the actual dynamic behavior of data, thus focusing on data characteristics which were strongly transient and for this reason probably not modeled in the off-line static analysis, which privileged extraction of static periodic or quasi-periodic behaviors. This method enabled us to obtain valuable results both in terms of accuracy and computational time. Its main disadvantage was that it was less able to characterize static aspects of the data.

A further consideration is needed related to the variability of monitoring performance. Three kinds of variability were investigated in the simulations. The first one was due to the stochastic nature of principal ICA algorithms, which caused different results to be obtained in different runs of the algorithm on the same data. Multiple repetitions of the analysis showed that this variability can affect computation time, but the obtained performance had a stability better than 10%. The second kind of variability identified was subject specific which caused about 20% of the variability. The third source of variability in the dynamic performance monitoring related to the specific target IC within a subject. Across different monitored ICs within the same subject, the results of Table 1 and Figure 2 confirm that the difference in behavior of different subjects was consistent across ICs. Indeed the performance improved for all the subjects when monitoring task related RVMT and LVMT (Figure 1) with respect to Resting State Network (RSN) related Default Mode Network (DMN) (Figure 2) thus proving that difference in the nature of monitored IC was the strongest source of variability (up to 30%) for these kind of presented methods. This may be due to the fact that different activations have particular statistical distribution properties, being more or less suitable to be extracted by ICA algorithms. In addition, a reason for the difficulty in extracting spatial characterization of the DMN is its low frequency relative to the sliding time window length, thus making it difficult for the algorithm to correctly follow it.

This work has some limitations. One limitation is related to the definition of dynamic monitoring performance, which depends on temporal or spatial correlations with a template reference derived from the whole time course. It is not necessarily correct to expect that spatial-temporal characteristics derived from the sliding window along the time course should match the ones derived from the whole time course. For this reason the performance measures are only indicative.

The possibility that the actual dynamic brain activation is correctly identified by these on-line methods opens the door to future definition of techniques and experiments. These experiments could exploit these methods to have a confirmation of transient activation identification independently of the off-line analysis, which represents a general reference for evaluation of results, but may also represent a bias.

Another limitation relates to the simulation nature of the work, which should be further evaluated on a real implementation in which the performance of the different methods can be studied, for example using a neurofeedback setup.

The comparison of ICA with non-ICA approaches in rt-fMRI setups was beyond the scope of this study. Given the known potential advantages of data-driven analysis (Norman et al., 2006; Magland et al., 2011; Sitaram et al., 2011), and in particular ICA methods (Esposito et al., 2003; Beckmann and Smith, 2004; Calhoun et al., 2009), this simulation study is limited to the comparison of novel ICA-based methods for rt-fMRI using robust activation in well-known visual motor areas. Future studies will be needed to evaluate these ICA methods with brain activation that could be more challenging to identify.

This work proposed and evaluated several strategies for using *a priori* information for the monitoring of brain networks in real-time fMRI experiments. The performance of the methods was characterized by both computation speed and correlation between the spatial-temporal properties of a target independent component derived dynamically and a reference component. The method that gave the highest performance was based on the Back-projection of a constant target spatial map derived by the spatial localizer. In this method the use of ICA was exploited only in the Functional Localizer phase, while during the on-line monitoring the reference component was kept constant and not updated with any ICA algorithm. This combination of both ICA and non-ICA methods shows thus to be very helpful and promising. This method had the limitation that its reference was constant and this means that it may not be optimal to follow dynamic changes as it cannot adapt to changes in brain. The other tested methods were based on the use of adaptive spatial, temporal, or spatial-temporal priors and may have useful applications in studies where there is a need of higher flexibility to monitor variable activation.

## Conclusion

This study proposed and evaluated several strategies for using spatial and/or temporal *a priori* information in ICA-based methods for the monitoring of brain networks in real-time fMRI experiments. The effectiveness of the novel real-time ICA-based method was evaluated against the off-line ICA analysis that is typically possible after all data has been acquired. The performance of the methods was characterized by both computation speed and correlation between the spatial-temporal properties of a target independent component derived dynamically and a reference component. In our testing conditions of relative low frequency task-induced activations (with a period of 20 s) we found that the Back-projection method outperformed the other methods giving the highest spatial-temporal correlations to the reference and the fastest computation time. The Back-projection method here investigated uses the ICA decomposition only in the functional localizer data, and not during the dynamic on-line analysis. It remains to be further investigated whether the spatial-temporal constrained methods can be better, as in principle expected, in situations where the networks to be monitored have higher frequency fluctuations in space and time.

## Conflict of Interest Statement

The authors declare that the research was conducted in the absence of any commercial or financial relationships that could be construed as a potential conflict of interest.
